# Routine Chest X-Rays Are Inaccurate in Detecting Relevant Intrapulmonary Anomalies During Medical Assessments of Fitness to Dive

**DOI:** 10.3389/fphys.2020.613398

**Published:** 2021-01-06

**Authors:** Thijs T. Wingelaar, Leonie Bakker, Frank J. Nap, Pieter-Jan A. M. van Ooij, Edwin L. Endert, Rob A. van Hulst

**Affiliations:** ^1^Diving Medical Center, Royal Netherlands Navy, Den Helder, Netherlands; ^2^Department of Anaesthesiology, Amsterdam UMC, Location AMC, Amsterdam, Netherlands; ^3^Woensdrecht Airbase, Royal Netherlands Airforce, Woensdrecht, Netherlands; ^4^Department of Radiology, Central Military Hospital, Ministry of Defence, Utrecht, Netherlands; ^5^Department of Pulmonology, Amsterdam UMC, Location AMC, Amsterdam, Netherlands

**Keywords:** fitness to dive, CXR, HRCT, choosing wisely, bullae, blebs, preventive medicine, occupational medicine

## Abstract

**Introduction:** Intrapulmonary pathology, such as bullae or blebs, can cause pulmonary barotrauma when diving. Many diving courses require chest X-rays (CXR) or high-resolution computed tomography (HRCT) to exclude asymptomatic healthy individuals with these lesions. The ability of routine CXRs and HRCT to assess fitness to dive has never been evaluated.

**Methods:** Military divers who underwent yearly medical assessments at the Royal Netherlands Navy Diving Medical Center, including CXR at initial assessment, and who received a HRCT between January and June 2018, were included. The correlations of CXR and HRCT results with fitness to dive assessments were analyzed using Fisher's exact tests.

**Results:** This study included 101 military divers. CXR identified bullae or blebs in seven divers, but HRCT found that these anomalies were not present in three subjects and were something else in four. CXR showed no anomalies in 94 subjects, but HRCT identified coincidental findings in 23 and bullae or blebs in seven. The differences between CXR and HRCT results were statistically significant (*p* = 0.023). Of the 34 subjects with anomalies on HRCT, 18 (53%) were disqualified for diving.

**Discussion:** Routine CXR in asymptomatic military divers does not contribute to the identification of relevant pathology in fitness to dive assessments and has a high false negative rate (32%). HRCT is more diagnostic than CXR but yields unclear results, leading to disqualification for diving. Fitness to dive tests should exclude routine CXR; rather, HRCT should be performed only in subjects with clinical indications.

## Introduction

Pulmonary barotrauma (PBT) is a serious adverse event in divers that can lead to (fatal) diving accidents (Russi, 1998; Vann et al., 2011). During diving, ambient pressure increases when divers descend and decreases when divers ascend, resulting in decreased and increased air volumes, respectively, as described by Boyle's Law (Bosco et al., 2018). Therefore, inadequate ventilation of (parts of) the lung can play an important role in the chain of events leading up to PBT and associated arterial gas embolism (Russi, 1998; Bosco et al., 2018). Anatomical or functional obstructions can prevent the alveoli from communicating with the outside world, leading to PBT (Tetzlaff et al., 1997; Russi, 1998). To assess the architectural aspects of the lungs and to determine the presence of obstruction, many pre-course fitness to dive screenings routinely include imaging of the thorax and pulmonary function tests (British Thoracic Society Fitness to Dive Group, 2003).

In diving medicine, chest X-rays (CXRs) are frequently used for imaging of the thorax, as they are widely available and have little impact on resources. Although technical advances have significantly improved the clarity of CXRs, the a priori likelihood of detecting a relevant lung disease that could result in air trapping or PBT when diving, such as extensive lung diseases and infectious diseases such as tuberculosis, is relatively low due to successful public health interventions and lower incidences of these diseases (World Health Organisation., 2016; Xie et al., 2020). Conversely, because the incidence of relatively rare conditions, such as lung cysts, bullae and blebs, are likely to have remained constant over the years, these conditions may be more readily identified due to the increased usage of CXR in general and in emergency medicine (Tigges et al., 2004; Plurad et al., 2007).

More advanced techniques, such as high-resolution computed tomography (HRCT), supply vastly more information than CXR, although they also involve exposure to higher doses of ionizing radiation (Plurad et al., 2007). It is unclear whether routine imaging has added value in evaluating fitness to dive, as routine CXR or HRCT frequently shows “incidental findings” when assessing fitness to dive (Levinson et al., 2015; Tan et al., 2020). Often these findings are of unknown clinical or medical significance but could result in a disqualification for diving, or prompt additional investigations or therapeutic interventions.

The lack of routine CXR during fitness to dive assessment may result in the under-detection of a pathologic condition, which, if identified, would have prevented a diving incident or fatal outcome (Lippmann et al., 2011). The relative advantages and disadvantages of the two possibilities, i.e., preventing possible harm by performing a routine CXR or HRCT vs. causing possible harm as a result of incidental findings of unknown medical relevance to diving and associated follow-up diagnostics, remain unclear, as relatively little is known in relation to either possibility in medical assessments of fitness to dive.

This retrospective study compared CXR and HRCT findings in asymptomatic military divers. We hypothesized that CXR would be less accurate than HRCT in detecting intrapulmonary pathology, and that HRCT would yield a high rate of incidental findings of unknown relevance and may result in medical disqualification for diving.

## Methods

The methods for handling personal details and privacy were in accordance with national and European legislation, as well as with the guidelines of the Association of Universities in the Netherlands.

### Data Collection

In compliance with international professional standards, military divers in the Netherlands undergo yearly medical assessments, including routine CXR (AP and lateral) at initial assessment, at the Royal Netherlands Navy Diving Medical Center (Wendling and Nome, 2004). Additionally, divers were referred to a military pulmonary specialist for HRCT imaging when spirometry showed a vital capacity >120% higher than ERS/ECSC-1993 reference values, even though a diver could have been free of symptoms of pulmonary disease [The latter practice has since been abandoned, as it was found to be inaccurate when using modern GLI-2012 reference values (Wingelaar et al., 2018; Wingelaar-Jagt et al., 2020)]. The records of all divers who underwent CXR and HRCT between January 2013 and June 2018 were included in this study.

CXR results were coded in a separate database as “no anomalies” or “bullae and blebs,” whereas HRCT results were coded as “no anomalies,” “bullae and blebs,” or “other findings.” A bleb was defined as an intrapulmonary, subpleural, thin-walled (<1 mm), air-containing space 10–20 mm in diameter, and a bulla was defined as a similar space >20 mm in diameter (Webb and Higgings, 2016). The result of the dive medical assessment was also added to this database.

### Analysis

All statistical analyses were performed with SPSS Statistics for Windows software (2015, version 23.0; IBM Corp; Armonk, NY), using Fisher's exact test for hypothesis testing. The alpha value was set at 0.05, and therefore statistical significance was defined as *p* < 0.05.

## Results

Between January 2013 and June 2018, 1,070 medical assessments of divers were performed, with 158 individuals referred to the pulmonary specialist. During 57 assessments, either no HRCT was performed, or the results could not be acquired for this study. Therefore, this study included 101 male military divers (average age, 36.4 yrs; range, 18–53 yrs) who underwent both CXR and HRCT. None of these divers had pulmonary complaints or a history of asthma, obstructive lung disease or bronchial hyperreactivity.

### Imaging Results

CXR detected bullae or blebs in seven (7%) divers, with no anomalies in the other 94 (93%). HRCT of three (43%) of the former seven divers showed no bullae or blebs, whereas HRCT of the other four (57%) yielded other anomalies, such as “density in the apical right region.” HRCT of the 94 subjects with no anomalies on CXR identified bullae or blebs in seven (7%) and other findings in 23 (24%), ranging from “physiological air trapping” and “bronchiectasis and paraseptal changes with no clinical significance” to “pericardial cyst” or “sarcoidosis.” The other findings could be differentiated into “pulmonary” anomalies in 13 subjects and “extrapulmonary” findings in 14 cases. None of the CXR findings of bullae or blebs could be confirmed on HRCT (i.e., no true positives in our sample). These results are summarized in Table 1. The difference between HRCT and CXR was statistically significant (*p* = 0.023 by Fisher's exact test), confirming the hypothesis that HRCT and CXR do not produce the same results.

**Table 1:** Results of CXR and HRCT.

		**HRCT**				
		**No abnormalities**	**Blebs or bullae**	**Other pulmonary findings**	**Other extrapulmonary findings**	**Total**
**CXR**	No abnormalities	64	7	13	10	94
	Blebs or bullae	3	0	0	4	7
	Total	67	7	13	14	101 (*p* = 0.023)

*CXR, chest X-ray; HRCT, high-resolution computed tomography*.

A bulla or bleb on CXR did not have any predictive value in this study (sensitivity of 0%), as none of the bullae or blebs seen on CXR could be confirmed on HRCT. Conversely, a CXR negative for bullae or blebs was incorrect in 11% of subjects (seven on HRCT vs. 64 “clean” CXRs), leading to a specificity of 90%. When all intra- and extrapulmonary abnormalities on HRCT were included, CXR was incorrect in 30 subjects (32%) compared with the 94 CXRs without any abnormalities.

### Fitness to Dive Assessment

Of the seven subjects with a bulla or bleb on HRCT, two were deemed fit to dive, whereas the other five (71%) were disqualified based solely on this finding (i.e., no other result from the medical assessment would have disqualified them). Of the 13 subjects with other pulmonary findings on HRCT, seven were disqualified (54%). Of the 14 subjects with extrapulmonary findings, seven (50%) passed the dive medical assessment. The other seven were disqualified based on the HRCT results, as well as on additional grounds, e.g., an insufficient cycle ergometry result (Table 2). Overall, the likelihood of being disqualified based on any abnormality found on HRCT was 53%. Of the 101 divers, 11 (11%) were disqualified based solely on HRCT findings.

**Table 2:** Associations between HRCT results and fitness to dive assessments.

**HRCT result**	**Qualified**	**Disqualified**	**Total**
Bleb or bulla	2 (29%)	5 (71%)	7
Other pulmonary findings	6 (46%)	7 (54%)	13
Extrapulmonary findings	7 (50%)	7 (50%)^*^	14
Total	16 (47%)	18 (53%)	34

* *In addition to the HRCT results, these seven subjects were disqualified on additional grounds*.

## Discussion

This study provides evidence that routine CXR in asymptomatic military divers as part of a medical assessment of fitness to dive does not contribute to the identification of intrapulmonary abnormalities, such as bullae or blebs, and has a high false negative rate (32%). Although HRCT was significantly more diagnostic than CXR, HCRT often yields findings of unclear medical significance regarding fitness to dive, with subjects having about a 50% likelihood of being disqualified for diving based solely on HRCT findings.

In many countries, including the Netherlands, CXR is mandatory during medical analysis of fitness to dive. Although the original rationale for including routine CXR in assessment of divers is not clear, it is included in aerospace medicine to detect diseases such as sarcoidosis and tuberculosis. Because the incidence of tuberculosis has markedly decreased in developed countries, however, CXR is no longer a cornerstone of its diagnosis (World Health Organisation., 2016). Additionally, it is unclear whether CXR is the ideal modality to detect intrapulmonary pathology, such as bullae or blebs. As this study showed, a comparison of CXR with HRCT results found that about one-third of CXRs showing no abnormalities were incorrect. Similarly, HRCT alone was shown to be a valid instrument to detect bullae or blebs in a preoperative setting (Kawaguchi et al., 2013). By contrast, CXR is insufficiently sensitive for screening. And while there are reports of gross pulmonary pathology detected by CXR in cases of cerebral arterial gas embolism (CAGE), these cases are extremely rare and perhaps do not justify CXR in every candidate diver (Weenink et al., 2012).

Although HRCT is regarded as the reference standard for detection of intrathoracic anomalies, it has resulted in overdiagnosis in many patients (Almajid et al., 2019). Moreover, post-mortem HRCT of 130 subjects without a history of pulmonary complaints or disease found small bullae in about one-third of these subjects (de Bakker et al., 2020), a finding attributed to the increased radiation used when performing post-mortem HRCTs. These small blebs would likely have been missed by HRCTs performed on living subjects. Although the military diving community is arguably different from the general population, the incidence of bullae or blebs is likely higher than previously thought. The relevance of these findings to fitness to dive is unclear, as the incidence of PBT is far lower than that of intrapulmonary abnormalities. To our knowledge there are no data on treatment of bullae or blebs or on the incidence of PBT in divers, but these treatments have proven ineffective in aviation medicine (Bang et al., 2019).

HRCT also showed findings of unknown medical significance for fitness to dive in a large percentage of our study subjects. These coincidental findings included “groundglass noduli” and “possible adhesions.” Findings such as “physiological air trapping” or “areas with low lung compliance” are common in the general healthy population and do not necessarily disqualify an individual for diving (Mets et al., 2012). Although about 50% of subjects with these coincidental findings were disqualified for diving, all subjects with anomalous findings required additional evaluations, increasing costs and delaying the result of the medical assessment and return to diving. Although this may increase safety, it was likely that the percentage of coincidental abnormalities was similar in the nearly 1,000 subjects who did not undergo HRCT during the period of this study. Moreover, there were few or no diving accidents in this population due to or including PBT, suggesting that these HRCT findings have limited clinical value.

Several recent studies have evaluated the contribution of routine tests, such as spirometry and audiometry, on medical assessment of fitness to dive (Voortman et al., 2016; Sames et al., 2018, 2019; Wingelaar et al., 2019). Overall, these studies suggest a more weighted approach to additional tests, such as whether subject history or physical examination is indicative of the need for further testing. Moreover, although divers have withheld critical information from physicians assessing fitness to dive, leading to diving incidents, the inclusion of a frequently inaccurate measure, such as CXR, or tests with unclear significance, such as HRCT, likely has limited additional value in healthy and fit subjects such as military divers (Lippmann and Taylor, 2020). In addition to increased costs and delays, these examinations result in greater exposure to radiation, as well as possible additional (invasive) medical examinations. Although recent advances in iterative CT reconstruction algorithms allow similar levels of details to HRCT, coupled with a level of exposure to radiation similar to that of CXR, these algorithms still provide coincidental findings of unknown clinical significance and suggest the need for additional investigations (Geyer et al., 2015). The results of the present study provide sufficient evidence to exclude routine CXR from initial medical assessment of fitness to dive. Rather, subjects should be through thoroughly assessed using history-taking and physical examination and referred to a pulmonary specialist to evaluate possible pulmonary disease when there are signs or symptoms. These results also suggest that, if imaging is indicated, HRCT should be performed, as CXR is not sensitive enough to exclude pathology such as bullae or blebs. The Royal Netherlands Navy has implemented these changes and now exclude routine CXR at initial assessment and perform HRCT when indicated.

### Strengths and Limitations

To our knowledge this is the first retrospective analysis comparing CXR and HRCT during medical assessment of fitness to dive in healthy, asymptomatic military divers. Although prospective studies might provide more valuable results, many nations have legislation limiting subject exposure to radiation. Therefore, a prospective study involving asymptomatic subjects undergoing both CXR and HRCT for research purposes only is unlikely. Additionally, subjects who know the results of the present study may be reluctant to participate due to the greater likelihood of being disqualified for diving. A blinded study, in which subjects undergo both examinations but the results are not known to the physician, could be regarded as unethical, as possible diagnoses might be missed and could contribute to accidents or injury. We feel that retrospective analyses are probably the highest achievable level of evidence on this subject. The agreement between our results and surgical findings suggests that our results are valid (Weenink et al., 2012; Kawaguchi et al., 2013).

This study had several limitations. First, the study population was highly selective, as healthy military divers are not necessarily representative of commercial and recreational divers. The a priori likelihood of pulmonary disease might differ in these populations, resulting in differences in their rates of detection by CXR and HRCT. However, these healthy and asymptomatic divers may be a more valid population to determine the accuracy of both modalities, as there are few confounding factors to affect the detection ratio. Second, this study does not include the results of pulmonary function tests (PFT). According to our old protocol, subjects with a vital capacity ≥120% higher than predicted by the ERS/ECSC-1993 guidelines were regarded as abnormal and referred to a pulmonary specialist. New insights gained from the GLI-2012 guidelines made these criteria obsolete (Wingelaar et al., 2018; Wingelaar-Jagt et al., 2020). Because almost all of our subjects had a PFT within 95% of normal (i.e., a *Z*-score of ±1.96), the subgroups would have been too small for accurate analysis. Moreover, the numbers of anomalies on CXR and HRCT were too small to divide into reliable subgroups based on smoking status. Although smoking increases the likelihood of spontaneous pneumothorax and its recurrence, its effect on the incidence of bullae and blebs remains unclear (Cheng et al., 2009). Although smoking has been linked to the development of emphysema, our target population was relatively young, suggesting that the effects of smoking in this population were likely below the limit of detection, especially for CXR. Physicians assessing fitness to dive should consider whether a PFT or smoking status should trigger additional imaging. Lastly, subjects did not undergo CXR and HRCT at the same point in time. Rather, subjects routinely underwent CXR prior to the medical assessment of fitness to dive, with HRCT always performed later. Thus, some of the abnormalities found on HRCT may not have been present at the time of CXR. Although this was unlikely, it may have affected the predictive value of CXR. However, several of the abnormalities found on HRCT, such as “groundglass noduli” and “possible atelectasis,” were unlikely to be detected by CXR, especially in the absence of clinical symptoms. Taken together, these findings indicate that CXR should not be used routinely to screen subjects for fitness to dive.

## Conclusion

This study showed that routine CXR to screen for subtle intrapulmonary pathology, such as bullae and blebs, is not reliable in asymptomatic military divers. Although HRCT detects more anomalies, many of these were of unknown medical significance in determining fitness to dive but could still lead to medical disqualification for diving. The authors suggest that initial medical assessments of fitness to dive should exclude routine CXR, whereas HRCT should be performed only in patients with clinical indications.

## Data Availability Statement

The raw data supporting the conclusions of this article will be made available by the authors, without undue reservation.

## Ethics Statement

Ethical review and approval was not required for the study on human participants in accordance with the local legislation and institutional requirements. Written informed consent for participation was not required for this study in accordance with the national legislation and the institutional requirements.

## Author Contributions

TW: conception and design of the study, analysis and interpretation of the data, and drafting and revising the manuscript. LB: conception and design of the study, acquisition and interpretation of the data, and drafting and revising the manuscript. FN: analysis and interpretation of the data, and revising the manuscript. P-JO and EE: interpretation of the data, and revising the manuscript. RH: design of the study, interpretation of the data, and revising the manuscript. All authors contributed to the article and approved the submitted version.

## Conflict of Interest

The authors declare that the research was conducted in the absence of any commercial or financial relationships that could be construed as a potential conflict of interest.
